# Somatic gene variation profiles in geriatric and adult malignant melanoma patients

**DOI:** 10.3389/fonc.2025.1649387

**Published:** 2025-10-10

**Authors:** Busra Ekinci, Ibrahim Halil Erdogdu, Seda Orenay-Boyacioglu, Olcay Boyacioglu, Nesibe Kahraman-Cetin, Dilara Akin, Merve Turan, Canten Tataroglu

**Affiliations:** ^1^ School of Medicine, Department of Medical Pathology, Aydin Adnan Menderes University, Aydin, Türkiye; ^2^ School of Medicine, Department of Medical Genetics, Aydin Adnan Menderes University, Aydin, Türkiye; ^3^ Faculty of Engineering, Aydin Adnan Menderes University, Aydin, Türkiye; ^4^ School of Medicine, Department of Oncology, Aydin Adnan Menderes University, Aydin, Türkiye

**Keywords:** genetic variants, geriatrics, malignant melanoma, NGS, somatic mutation

## Abstract

**Introduction:**

Skin cancer is a highly heterogeneous disease affecting substantial geriatric individuals. Therefore, understanding gene variants and their presence in geriatric and adult skin cancer patient groups is valuable for the improvement of healthcare policies. The somatic variation profile in geriatric patients diagnosed with malignant melanoma (MM) was examined retrospectively by comparing them to the younger cases to reveal the clinical importance of the panel tests.

**Methods:**

The study included all adult MM patients referred to Molecular Pathology Laboratory from Oncology Clinic between 2019 and 2023. The patients (*n* = 103) were chronologically divided into geriatric (≥65) and adult (<65 years) groups. The results of targeted next generation sequencing panel studied with probe-capture method were evaluated retrospectively.

**Results:**

Among the study cohort, 58 (56.31%) were male, 45 (43.69%) were female, and also 55 were in the geriatric age group, 48 were in the adult group with an overall mean age of 63.30 years. The most commonly encountered pathogenic variants in the geriatric MM group were *BRAF* V600E (14.55%) and V600K (7.27%) variants in Exon 15 followed by *NRAS* (9.09%), *NF1* (9.09%), *KIT* (5.45%), *KRAS* (5.45%), *CDKN2A* (3.64%), and *PTEN* (3.63%). In the adult MM group, the most common pathogenic variants were *BRAF* V600E (39.58%) and V600K (8.33%) followed by *NRAS* (14.58%), *NF1* (8.33%), *PTEN* (8.33%), *BRCA2* (8.33%), and *TP53* (4.17%).

**Conclusions:**

Delineating the distribution of somatic variations in geriatric MM cases holds significant importance in the development of healthcare policies. These data are the first reported findings from Türkiye.

## Introduction

Malign melanoma (MM), although the rarest (2%) among other skin cancers, is one of the most extensively researched cancer types today due to its poor prognosis, increasing incidence, and mortality rates (1, 2). Being a malignant tumor of melanocytes and nevus cells, MM primarily originates in the skin, but can rarely arise from mucous membranes, meninges, eyes, and internal organs as well (1–4). In 2024, the estimated number of new melanoma cases is 100,640, with an estimated mortality rate of 8,290 in both sexes combined (5). Similar to many other cancer types, it is believed that environmental and genetic factors play a combined role in MM development. The main risk factors generally associated with MM development include skin type (fair skin), environmental factors (exposure to ultraviolet radiation), genetic background (*CDKN2A* gene mutation), pre-existing number of melanocytic nevi, presence of dysplastic nevi, and a history of previous melanoma (6).

In the present day, the understanding of melanomagenesis has been enhanced by the identification of signaling molecules and pathways involved in melanocyte development. Signaling pathways can be categorized into two groups: those affected by germline mutations and those by somatic mutations. The CDKN2A(p16)/CDK4/RB pathway and BAP-1 are the main regions affected by germline mutations. The majority of molecular events in melanoma development are driven by somatic mutations. The most commonly observed somatic mutation is a point mutation in the *BRAF* gene. Studies have shown that approximately 80% of *BRAF* point mutations detected in nearly 50% of melanomas result from the 1796T>A missense mutation, leading to the V600E amino acid alteration (6, 7).

More than 40% of MM cases are diagnosed in individuals aged 65 and older, often presenting distinct clinicopathological features. Geriatric patients are frequently diagnosed with advanced stages of the disease, characterized by larger Breslow thickness, increased ulceration frequency, and elevated mitotic index, which are well-defined negative prognostic factors. Advanced age is considered an independent poor prognostic factor in melanoma, considering its association with the aforementioned features. Unlike the improvement in mortality seen in young adults with MM over the past thirty years, mortality in older adults has remained stable. This discrepancy is thought to be due to potential differences in biological and molecular profiles (4, 6). However, while there is limited literature on the somatic gene mutation profile in geriatric cases of MM from other countries, there is currently no data available from Türkiye. Therefore, this study aims to compare the somatic mutation profile in geriatric patients diagnosed with MM to that of young adult cases and to elucidate the importance of somatic mutation profiling in geriatric patients.

## Materials and methods

### Ethical approval

The study obtained approval from the Institutional Non-Interventional Clinical Research Ethics Committee (protocol code: 223 and date of approval: 7 Dec 2023). The study was conducted by adhering to the criteria of the Helsinki Declaration.

### Cases

A total of 103 adult MM patients referred to the Molecular Pathology Laboratory from the external Oncology Clinic between January 2019 and November 2023 were included in the study cohort. Patients with undetermined diagnoses during screening were excluded. Gender, diagnostic subgroup, and age on diagnosis of the patients were documented, and all information was extracted from the electronic database of Molecular Pathology laboratory utilized for patient monitoring. The obtained data were grouped chronologically for Next Generating Sequencing (NGS) panel analyses. Accordingly, individuals aged between 18 and 64 were considered young adults, while those aged 65 and above were classified as geriatric patients.

### Tissue processing, preservation, and histopathological verification

Melanoma specimens, obtained by biopsy or surgical excision, were received from external Oncology centers for molecular testing as formalin-fixed, paraffin-embedded (FFPE) tissue blocks, which included both primary and metastatic samples. Hematoxylin and eosin (H&E)-stained sections prepared from these blocks were independently reviewed by two pathologists. Histopathological evaluation confirmed the diagnosis of MM, and the presence of tumor tissue was verified in the selected blocks. Additionally, detailed information on the primary tumor localization was not available for every case in our study. Therefore, we were unable to perform stratification based on the primary site in our analyses. Molecular analyses were performed only on samples with a tumor cell content greater than 50%. FFPE blocks were stored at room temperature in a dry, dark environment until DNA isolation.

### NGS and data analysis

DNA was isolated from these sections using the QIAamp DNA FFPE Tissue Kit (Qiagen, Hilden, Germany) according to the manufacturer's instructions. During DNA isolation, additional procedures were applied to minimize DNA fragmentation. The integrity of the DNA samples was assessed using a NanoDrop spectrophotometer (Thermo Fisher Scientific, Waltham, MA, USA) and samples with A260/280 ratios falling within the range of 1.8 to 2.0 were deemed suitable for inclusion in the study.

In all patients, all exons (including 5 bp of intron regions) of the 142 genes included in the QIAseq Expanded Cancer NGS Panel (Qiagen, Hilden, Germany) were sequenced after library preparation and barcoding using the NextSeq 550 platform ((Illumina, San Diego, CA, ABD). The full list of genes in the panel, along with their functional classifications (e.g., kinase, transcription factor, tumor suppressor, etc.), is provided in **Supplementary Table 1**. The quality of DNA libraries was quantitatively assessed using the Qubit dsDNA BR Assay system (Invitrogen, Carlsbad, CA, USA).

Sequencing data were analyzed using NextSeq software, and quality indicators were assessed through QCI (Qiagen Clinical Insight) analysis. These analyses ensured data reliability by evaluating key metrics such as read depth, coverage uniformity, and base quality scores. The targeted sequencing on the NextSeq 550 platform was designed to achieve an average coverage depth of 500X to ensure reliable detection of somatic variants. Since our study is a retrospective cohort, no specific control group was included; however, the results were validated by comparison with reference sequences in the literature and previous studies.

Variant selection and classification were performed independently using both Clinical Insight and Ingenuity software (Qiagen, Hilden, Germany). This dual-software approach allowed accurate identification of pathogenic, likely pathogenic, variants of uncertain significance, likely benign, and benign variants. All identified variants were visually inspected using IGV 2.8.2 software to minimize potential errors from automated calling. This step also helped filter out possible technical artifacts and low-quality calls. Finally, all variants were categorized according to clinical significance and potential target drugs based on the guidelines of the Association for Molecular Pathology (AMP), College of American Pathologists (CAP), American Society of Clinical Oncology (ASCO), and American College of Medical Genetics and Genomics (ACMG).This multi-step bioinformatics pipeline, combining automated variant calling, visual verification, and library quality controls, ensured both the reliability and reproducibility of the results.

### Statistical analysis

Numerical data were provided as mean ± standard deviation. A p-value of less than 0.05 was considered statistically significant. Chi-square, Fisher`s exact, t-test, and ANOVA test were used for statistical evaluation of the data.

## Results

Out of the 103 patients included in the analysis, 58 (56.31%) were male and 45 (43.69%) were female, with a mean age of 63.30 ± 5.89. Also, 55 patients were in the geriatric age group (≥65 years) and 48 were in the adult (<65 years) group. The most common metastases observed in patients were lymph node, soft tissue, and brain metastases.

Geriatric MM group included 33 males and 22 females. When the metastatic status of geriatric MM patients was observed, lymph node metastasis was detected in 10.91% of patients, soft tissue metastasis in 9.09%, liver metastasis in 7.27%, brain metastasis in 3.64%, and various types of metastases including skin, lung, tibia, abdomen, and parotid regions in 1.88% of patients.

In the adult MM group, there were 25 males and 23 females. When the metastatic status was observed in this group, lymph node metastasis was found in 25% of patients, brain metastasis in 8.33%, soft tissue metastasis in 6.25%, liver and lung metastasis in 4.17% each, and various types of metastases including sacrum, breast, and bone regions in 2.08% of patients. Metastases were more common in the geriatric MM group than the adult MM group. When the two groups were compared in terms of organ metastases, no statistically significant difference was observed (p > 0.05). The clinopathological features of the patients are shown in **Table 1**.

**Table 1:** Clinicopathological findings in adult and geriatric MM patients.

Clinicopathological findings	Adult MM group (n/%)	Geriatric MM group (n/%)	P value
Number of patients	48	55	
Average age	53.8 ± 7.2	70.4 ± 4.5	
Gender			0.067
Male	25 (52.00%)	33 (60.00%)	
Female	23 (48.00%)	22 (40.00%)	
Metastatic areas			
Lymph node metastasis	12 (25.00%)	6 (10.91%)	0.061
Brain metastasis	4 (8.33%)	2 (3.64%)	0.056
Liver metastasis	2 (4.17%)	4 (7.27%)	0.114
Soft tissue metastasis	3 (6.25%)	5 (9.09%)	0.718
Other organ metastasis	1 (2.08%)	1 (1.88%)	0.980

**Figure 1:**
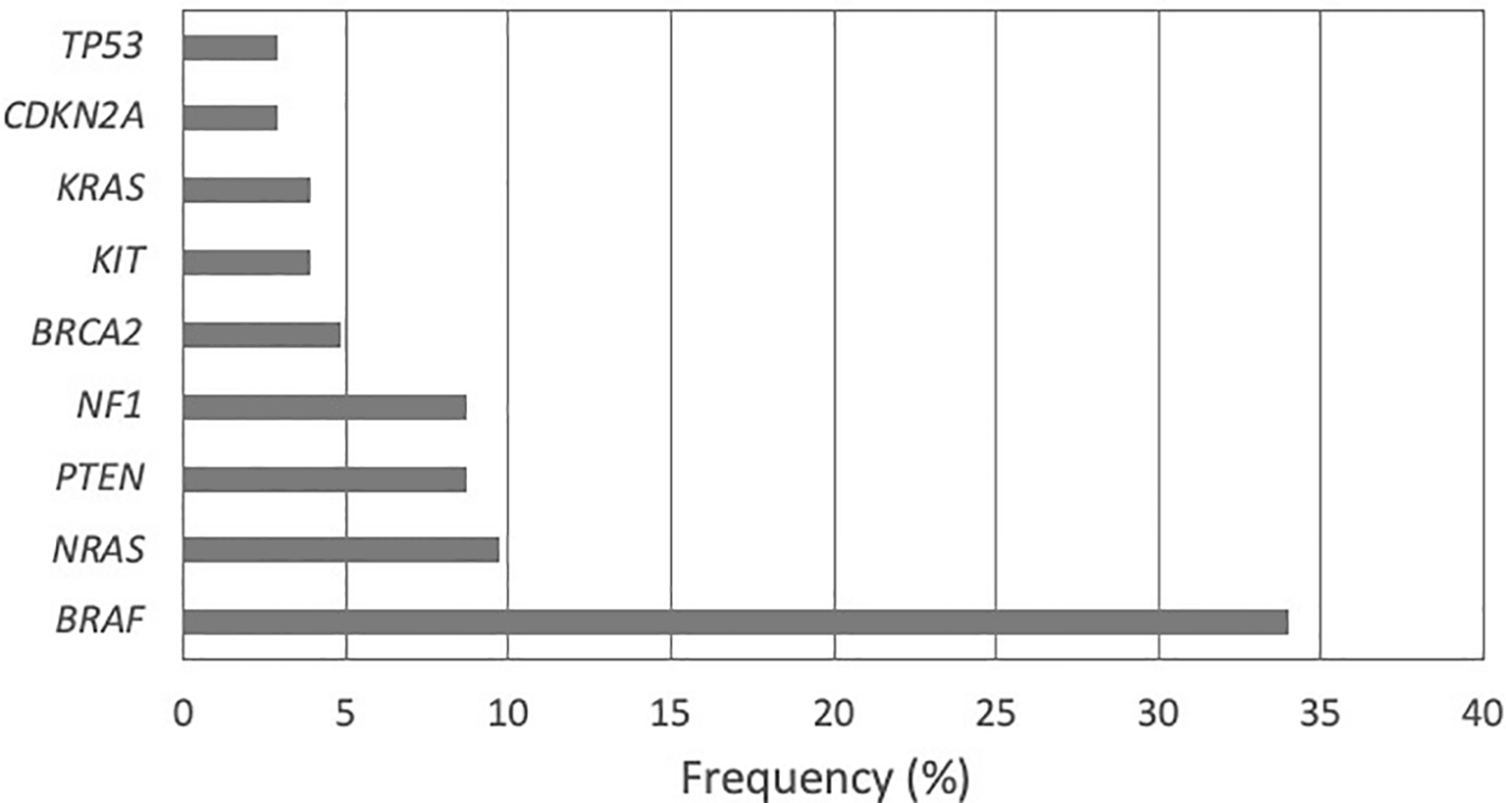
Frequency of pathogenic variants detected in 142-gene panel among patients with MM.

The gene panel analysis containing 142 genes demonstrated 48 different pathogenic variations in 63 patients (61.17%). The number of patients with no pathogenic variations detected was 40 (38.83%). The most frequent pathogenic variations were found in the *BRAF* gene (33.98%) followed by *NRAS* (9.71%), *PTEN* and *NF1* (8.74% each), *BRCA2* (4.85%), *KIT* and *KRAS* (3.88% each), *CDKN2A* (2.91%), and *TP53* (2.91%) (**Figure 1**).

The comparative analysis of pathogenic variants between geriatric and adult MM patients revealed age-related differences in their molecular profiles. In geriatric MM patients, the most frequently observed variants were *BRAF V600E* (14.55%) and *V600K* (7.27%) in Exon 15, followed by *NRAS* (10.90%), NF1 (9.09%), *KIT* and *KRAS* (5.45% each), *CDKN2A* (3.64%), and *PTEN* (3.63%). In adult MM patients, *BRAF V600E* (39.58%) and *V600K* (8.33%) were again the most common, followed by *NRAS* (14.58%), *NF1, PTEN*, and *BRCA2* (8.33% each), and *TP53* (4.17%). Statistical analysis indicated that *BRAF V600E* (p = 0.02) and *BRCA2* (p = 0.05) were significantly more frequent in adults, while other variants did not differ significantly between age groups. Overall, these findings suggest that *BRAF V600E* and *BRCA2* mutations are more characteristic of adult MM, whereas other variants are more evenly distributed across age groups. The ten most frequently observed variants in geriatric and adult patients are summarized in **Table 2**.

**Table 2:** Frequency of pathogenic variants in geriatric and adult MM patients.

Gene/variant	Geriatric MM (n/%)	Adult MM (n/%)	P value (fisher`s exact)
*BRAF V600E*	8 (14.55%)	19 (39.58%)	0.02^*^
*BRAF V600K*	4 (7.27%)	4 (8.33%)	1.00
*NRAS*	6 (10.90%)	7 (14.58%)	0.77
*NF1*	5 (9.09%)	4 (8.33%)	1.00
*KIT*	4 (8.00%)	1 (2.00%)	0.12
*KRAS*	3 (5.45%)	0 (0.00%)	0.25
*CDKN2A*	3 (5.00%)	1 (2.08%)	0.63
*PTEN*	2 (3.63%)	4 (8.33%)	0.40
*MAP2K1*	3 (5.45%)	0 (0.00%)	0.25
*CHECK2*	3 (5.45%)	0 (0.00%)	0.25
*TP53*	2 (3.64%)	2 (4.17%)	0.85
*BRCA2*	0 (0.00%)	4 (8.33%)	0.05^*^
*PIK3CA*	0 (0.00%)	2 (4.17%)	0.23
*GNAQ*	0 (0.00%)	1 (2.08%)	0.45

*Significant p < 0.05.

## Discussion

Age serves as a notable prognostic indicator for individuals diagnosed with melanoma, with distinct clinical presentations and disease progression observed in elderly patients compared to younger ones (8). In our study, similar to the literature, more metastases and somatic variants were observed in geriatric patients, supporting a poor prognosis.

In this study, the most frequently detected mutations across both groups were *BRAF* variants (33.98%), followed by *NRAS* (11.65%), *PTEN* (8.74%), and *NF1* (8.74%) variants. The *BRAF* gene is responsible for encoding a serine/threonine protein kinase that plays a crucial role in regulating the Ras/Raf/MEK/ERK signaling pathway. *BRAF* mutations, which are found in about 60% of melanomas, were initially identified in 2002 in human malignancies. The V600E, the most prevalent mutation, accounts for 80% of primary melanomas and leads to the structural activation of MEK (9). The next two common mutations in *BRAF* gene are V600K (20%) and V600R (7%). Typically, *BRAF* mutations are linked to melanomas detected in young individuals and areas intermittently exposed to sunlight (10–14). In our study, *BRAF* mutations were predominantly identified in metastatic adult MM patients. This observation aligns with existing literature and substantiates the notion of a more aggressive phenotype in melanomas harboring *BRAF* mutations, even in thin lesions.

*NRAS* mutations in melanoma patients are observed at rates of approximately 13-25% (15). In clinical practice, *NRAS* mutations are frequently linked to elderly patients (age > 55) and individuals with chronic UV radiation exposure. These mutations correlate with a poorer prognosis, characterized by elevated rates of visceral and CNS metastases (10, 15). Consistent with the literature, in our study, *NRAS* variants were observed in MM patients at a rate of 9.71%. However, contrary to expectations, *NRAS* variants were more frequently observed in the adult group.

In melanoma patients, several additional genetic mutations have been observed in the absence of currently available targeted therapeutic agents. *NF1* gene mutations, leading to the abrogation of negative regulatory pathways in RAS-associated MAPK pathways, exhibit a substantial mutation prevalence of 12-18% in melanoma patients. This rate escalates to 45-93% in desmoplastic histologic subtypes, positioning *NF1* mutations as the third most frequently observed driver mutation after *BRAF* and *NRAS* mutations (16). Consistent with the literature, *NF1* mutations were observed in our study as the third most common mutation after *BRAF* and *NRAS* variants, with a prevalence of 8.74% among all MM patients.

Tumor suppressor gene *PTEN* mutations are observed to be co-expressed with *BRAF* mutations in patients harboring *BRAF* mutations, contributing to immune escape of tumor cells through various mechanisms unrelated to the MAPK pathway, including downregulation of the tumor microenvironment and antitumoral immune cells within tertiary lymphoid structures. This contributes to suboptimal responses among certain patient groups receiving standard immunotherapies and targeted therapies (17). In our study, *PTEN* variants were observed in 8.74% of MM patients, and these variants particularly showed co-occurrence with *BRAF* mutations in geriatric patients, consistent with the literature.

The most common pathogenic mutations in melanoma reside in *BRAF, NRAS*, and *TP53* genes. However, essential genetic alterations have also been located in other genes such as *CDKN2A, KIT, GNAQ*, and *GNA11* (12, 18). The variations of the mutation frequencies in the above-mentioned genes result from the diverse genetic background of individuals and pathological characteristics of MM, delineating an intricate mutation model that holds promise for assessing the risk of metastasis development (19). In our study, in line with the literature, *KIT* variants were detected in 3.88% and *CDKN2A* variants in 2.91% of our MM patients, and these variations were more commonly observed in metastatic geriatric patients.

A recent whole exome sequencing study showed that the frequency of *TP53* mutations in melanoma is 19.0% (20). The current study observed two different *TP53* variants in 2.91% of overall MM cases. Until now, conflicting data have emerged concerning the significance of *TP53* mutations in melanoma (21). Additional studies are warranted to shed light on the *TP53* role in the progression of melanoma.

In the literature, both *KRAS* and *HRAS* mutations have been reported in approximately ~2% of melanomas (22). In our study, *KRAS* variants were observed at a similar rate of 3.98%, consistent with the literature, and were more commonly observed in geriatric patients.

Germline mutations in *BRCA1* and *BRCA2* significantly increase the susceptibility to both breast and ovarian cancer, as well as other cancers including pancreatic and prostate cancers. *BRCA2* mutation carriers have been observed to have elevated risks for both uveal and cutaneous melanomas (23). While there is limited research on the germline mutations of the *BRCA2* gene in melanoma, there is no information available regarding somatic variations. In our study, somatic *BRCA2* variations were detected in 4.85% of MM patients, suggesting that somatic mutations in *BRCA2* may also play a role in MM pathogenesis.

The primary melanoma site—chronic sun-damaged skin, non-sun-damaged skin, acral, mucosal, or other—is an important confounder of the mutational spectrum. Classic studies, including Curtin et al., have shown that *BRAF, NRAS, KIT, NF1*, and other alterations differ markedly by site. In our study, detailed information on the primary tumor localization was not available for every case (24, 25). Without this information, it is difficult to interpret whether age-related differences reflect tumor site distribution or age-related biology.

The findings of our study are generally consistent with previous large sequencing cohorts. These results align with data from the The Cancer Genome Atlas (TCGA) study, which reported *BRAF* mutations in approximately 50% of melanoma cases, *NRAS* mutations in 30%, *NF1* mutations in 12–23%, and *PTEN* mutations in 5–40% of cases (22, 26). Similarly, Hodis et al. (2012) identified *BRAF* mutations in 50% of melanomas, *NRAS* mutations in 30%, *NF1* mutations in 12–23%, and *PTEN* mutations in 5–40%, highlighting their role in melanoma progression and therapeutic responsiveness (20). Although the frequencies of *BRAF* and *NRAS* mutations in our cohort are slightly lower than those reported in these large-scale studies, the overall pattern of mutation prevalence is consistent. The high frequency of *BRAF* and *NRAS* mutations in particular provides important insights into the molecular heterogeneity of melanomas and potential sensitivity to targeted therapies, while *NF1* and *PTEN* mutations further illustrate the diversity of driver alterations in this population (27). Furthermore, the observed age-related differences in mutation profiles have important implications for the clinical management and therapeutic strategies of melanoma patients. In our cohort, geriatric patients exhibited higher frequencies of *NRAS, KIT, KRAS, CDKN2A*, and *PTEN* mutations, whereas *BRAF V600E* and *V600K* mutations were more prevalent in adult patients. These molecular distinctions suggest that older patients may benefit from tailored therapeutic approaches that consider both the type of driver mutations and potential differences in treatment response. Previous studies have reported that *NRAS* and *PTEN* alterations are associated with poorer prognosis and may influence the efficacy of targeted and immunotherapeutic interventions (20, 22, 26). Integrating such age-specific molecular information into clinical decision-making could therefore optimize outcomes and support the development of personalized management strategies for melanoma patients across different age groups.

Our study focuses on a clinically significant patient group (adult and geriatric melanoma patients) and utilizes a broad targeted NGS panel covering 142 genes. In addition, it provides a unique and valuable dataset as the first somatic gene variation profile of melanoma patients in Türkiye stratified by age. Age-related molecular differences (*BRAF, NRAS, KIT, KRAS, CDKN2A, PTEN*) were identified in the study, further emphasizing the clinical and molecular significance of the findings. Additionally, the observed age-related differences in mutation profiles have important implications for the management and therapeutic strategies of elderly melanoma patients. Our findings suggest that certain mutations, such as *NRAS, KIT, KRAS, CDKN2A*, and *PTEN*, are more prevalent in geriatric patients, which may influence disease progression and response to therapy. Highlighting these differences reinforces the need for age-specific considerations in clinical decision-making and supports the development of tailored treatment approaches that account for the molecular characteristics associated with patient age. However, the study has some limitations. The sample size is relatively small, which limits the generalizability of the results. Information on the primary tumor localization was not available for all cases, and sequencing data from metastatic and primary tissues were analyzed together, preventing complete assessment of tissue-specific effects. Moreover, the impact of age on mutation profiles was evaluated without adjustment for tumor site or stage. Future studies with larger, multicenter cohorts are warranted to enhance the generalizability of the findings and strengthen the prognostic and predictive value of the genetic profile.

## Data Availability

The data presented in the study are deposited in the Figshare repository, https://doi.org/10.6084/m9.figshare.30293974.
