# Supplementary material for: Somatic gene variation profiles in geriatric and adult malignant melanoma patients
Source: Front Oncol. 2025 Oct 10;15:1649387. doi: 10.3389/fonc.2025.1649387 (PMC12549232; doi:10.3389/fonc.2025.1649387)
Supplement: Supplementary file 1 [file Table1.docx]

**Supplementary Table 1.** Genes included in the NGS panel with their functional classification (e.g., kinase, transcription factor, tumor suppressor, etc.)

| ***Gene*** | ***Classifier (Kinase, Transcription Factor, Tumor Suppressor, etc.)*** |
| --- | --- |
| *ADA* | Enzyme / Metabolic Gene |
| *ANKRD26* | Scaffold Protein / Regulatory |
| *ASXL1* | Epigenetic Regulator / Tumor Suppressor |
| *ASXL2* | Epigenetic Regulator / Tumor Suppressor |
| *ATM* | Kinase / DNA Damage Response |
| *ATRX* | Chromatin Remodeler / Tumor Suppressor |
| *BCL6* | Transcription Factor |
| *BCOR* | Transcription Corepressor |
| *BCORL1* | Transcription Corepressor |
| *BCR* | Signaling / Kinase Pathway |
| *BIRC3* | Inhibitor of Apoptosis / Signaling |
| *BLM* | DNA Helicase / DNA Repair |
| *BRAF* | Kinase / MAPK Pathway |
| *BRCA1* | Tumor Suppressor / DNA Repair |
| *BRCA2* | Tumor Suppressor / DNA Repair |
| *C17orf97* | Unknown Function |
| *CARD11* | Scaffold Protein / Signaling |
| *CALR* | Chaperone / Endoplasmic Reticulum |
| *CBLC* | Adapter Protein / Signaling |
| *CDKN2A* | Tumor Suppressor / Cell Cycle Regulation |
| *CEBPA* | Transcription Factor / Hematopoiesis |
| *CHEK2* | Kinase / DNA Damage Response |
| *CREBBP* | Transcription Coactivator / Epigenetic Regulation |
| *CRLF2* | Cytokine Receptor / Signaling |
| *CSF1R* | Receptor Tyrosine Kinase / Hematopoiesis |
| *CSF3R* | Receptor Tyrosine Kinase / Hematopoiesis |
| *CTCF* | Transcription Factor / Chromatin Insulator |
| *CUX1* | Transcription Factor / Development |
| *DAXX* | Transcription Coactivator / Apoptosis |
| *DDX41* | RNA Helicase / Immune Response |
| *DNM2* | Dynamin / Endocytosis |
| *DNMT1* | DNA Methyltransferase / Epigenetic Regulation |
| *DNMT3A* | DNA Methyltransferase / Epigenetic Regulation |
| *EED* | Polycomb Group Protein / Epigenetic Regulation |
| *EGFR* | Receptor Tyrosine Kinase / Cell Signaling |
| *ELANE* | Protease / Neutrophil Function |
| *EP300* | Transcription Coactivator / Epigenetic Regulation |
| *ETV6* | Transcription Factor / Hematopoiesis |
| *EZH2* | Polycomb Group Protein / Epigenetic Regulation |
| *FAM154B* | Unknown Function |
| *FAM47A* | Unknown Function |
| *FAM5C* | Unknown Function |
| *FAS* | Cell Surface Receptor / Apoptosis |
| *FBXW7* | Ubiquitin Ligase / Tumor Suppressor |
| *FLRT2* | Cell Adhesion Molecule / Development |
| *FLT3* | Receptor Tyrosine Kinase / Hematopoiesis |
| *GATA1* | Transcription Factor / Hematopoiesis |
| *GATA2* | Transcription Factor / Hematopoiesis |
| *GJB3* | Gap Junction Protein / Cell Communication |
| *GNAS* | GTPase / Signal Transduction |
| *HNRNPK* | RNA Binding Protein / Splicing |
| *HRAS* | GTPase / Cell Signaling |
| *IDH1* | Enzyme / Metabolic Pathway |
| *IDH2* | Enzyme / Metabolic Pathway |
| *IKZF1* | Transcription Factor / Immune System |
| *IKZF3* | Transcription Factor / Immune System |
| *JAK1* | Kinase / Cytokine Signaling |
| *JAK2* | Kinase / Hematopoiesis |
| *JAK3* | Kinase / Immune System |
| *KAT6A* | Histone Acetyltransferase / Epigenetic Regulation |
| *KCNA4* | Potassium Channel / Ion Transport |
| *KCNK13* | Potassium Channel / Ion Transport |
| *KDM6A* | Histone Demethylase / Epigenetic Regulation |
| *KDR* | Receptor Tyrosine Kinase / Angiogenesis |
| *KIT* | Receptor Tyrosine Kinase / Hematopoiesis |
| *KLHDC8B* | Unknown Function |
| *KLHL6* | Unknown Function |
| *KMT2A* | Histone Methyltransferase / Epigenetic Regulation |
| *KRAS* | GTPase / Cell Signaling |
| *LRRC4* | Cell Adhesion Molecule / Nervous System |
| *LUC7L2* | RNA Binding Protein / Splicing |
| *MAP2K1* | Kinase / MAPK Pathway |
| *MLH1* | DNA Mismatch Repair / Tumor Suppressor |
| *MPL* | Receptor / Hematopoiesis |
| *MSH6* | DNA Mismatch Repair / Tumor Suppressor |
| *MYC* | Transcription Factor / Cell Cycle Regulation |
| *MYD88* | Adapter Protein / Immune Response |
| *NBN* | DNA Repair Protein / Tumor Suppressor |
| *NF1* | Tumor Suppressor / Ras Pathway |
| *NOTCH1* | Receptor / Developmental Pathway |
| *NPAT* | Transcription Factor / Cell Cycle Regulation |
| *NPM1* | Nucleolar Protein / Tumor Suppressor |
| *NRAS* | GTPase / Cell Signaling |
| *NSD1* | Histone Methyltransferase / Epigenetic Regulation |
| *NTRK3* | Receptor Tyrosine Kinase / Neurotrophin Signaling |
| *OR13H1* | Olfactory Receptor / Sensory Function |
| *OR8B12* | Olfactory Receptor / Sensory Function |
| *P2RY2* | Purinergic Receptor / Signal Transduction |
| *PAX5* | Transcription Factor / B-cell Development |
| *PCDHB1* | Cell Adhesion Molecule / Nervous System |
| *PDGFRA* | Receptor Tyrosine Kinase / Cell Signaling |
| *PHF6* | Transcription Factor / Hematopoiesis |
| *PIK3CA* | Kinase / PI3K Pathway |
| *PIK3R2* | Regulatory Subunit / PI3K Pathway |
| *PIM1* | Kinase / Cell Cycle Regulation |
| *PIM2* | Kinase / Cell Cycle Regulation |
| *PIM3* | Kinase / Cell Cycle Regulation |
| *PML* | Tumor Suppressor / Transcription Factor |
| *PRAMEF2* | Unknown Function |
| *PRF1* | Cytotoxic Protein / Immune Response |
| *PRPF40B* | Splicing Factor / RNA Processing |
| *PRPF8* | Splicing Factor / RNA Processing |
| *PSMA1* | Proteasome Subunit / Protein Degradation |
| *PTEN* | Tumor Suppressor / Cell Cycle Regulation |
| *PTPN11* | Phosphatase / Cell Signaling |
| *RAD21* | Cohesin Complex / Chromosome Segregation |
| *RANX1* | Transcription Factor / Hematopoiesis |
| *RB1* | Tumor Suppressor / Cell Cycle Regulation |
| *RELN* | Extracellular Matrix Protein / Nervous System |
| *RUNX1* | Transcription Factor / Hematopoiesis |
| *SETBP1* | Transcription Factor / Hematopoiesis |
| *SF1* | Transcription Factor / Splicing |
| *SF3A1* | Splicing Factor / RNA Processing |
| *SF3B1* | Splicing Factor / RNA Processing |
| *SH2B3* | Adapter Protein / Immune System |
| *SH2D1A* | Adapter Protein / Immune System |
| *SMARCB1* | Chromatin Remodeling / Tumor Suppressor |
| *SMC1A* | Cohesin Complex / Chromosome Segregation |
| *SMC3* | Cohesin Complex / Chromosome Segregation |
| *SRSF2* | Splicing Factor / RNA Processing |
| *SRP72* | Splicing Factor / RNA Processing |
| *STAG2* | Cohesin Complex / Chromosome Segregation |
| *STAT3* | Transcription Factor / Cytokine Signaling |
| *STXBP2* | Exocytosis Protein / Immune System |
| *SUZ12* | Polycomb Group Protein / Epigenetic Regulation |
| *TAL1* | Transcription Factor / Hematopoiesis |
| *TERC* | RNA Component / Telomerase |
| *TERT* | Enzyme / Telomerase |
| *TET2* | DNA Demethylase / Epigenetic Regulation |
| *TGFBR2* | Receptor / TGF-β Signaling |
| *TNFRSF13B* | Receptor / Immune System |
| *TP53* | Tumor Suppressor |
